# Implication of nanotechnology to reduce the environmental risks of waste associated with the COVID-19 pandemic

**DOI:** 10.1039/d3ra01052j

**Published:** 2023-04-20

**Authors:** Gharieb S. El-Sayyad, Dounia Elfadil, Mohamed S. Gaballah, Dina M. El-Sherif, Mohamed Abouzid, Hanady G. Nada, Mohamed S. Khalil, Mohamed A. Ghorab

**Affiliations:** a Department of Microbiology and Immunology, Faculty of Pharmacy, Ahram Canadian University (ACU) Giza Egypt Gharieb.Elsayyad@acu.edu.eg; b Department of Microbiology and Immunology, Faculty of Pharmacy, Galala University New Galala City Suez Egypt Gharieb.Elsayyad@gu.edu.eg; c Drug Microbiology Laboratory, Drug Radiation Research Department, National Center for Radiation Research and Technology (NCRRT), Egyptian Atomic Energy Authority (EAEA) Cairo Egypt Gharieb.S.Elsayyad@eaea.org.eg; d Biology and Chemistry Department, Hassan II University of Casablanca Morocco; e College of Engineering (Key Laboratory for Clean Renewable Energy Utilization Technology, Ministry of Agriculture), China Agricultural University Beijing 100083 PR China; f National Institute of Oceanography and Fisheries (NIOF) Cairo Egypt; g Department of Physical Pharmacy and Pharmacokinetics, Faculty of Pharmacy, Poznan University of Medical Sciences Rokietnicka 3 St. 60-806 Poznan Poland; h Doctoral School, Poznan University of Medical Sciences 60-812 Poznan Poland; i Agricultural Research Center, Central Agricultural Pesticides Laboratory Alexandria Egypt; j Wildlife Toxicology Laboratory, Department of Animal Science, Institute for Integrative Toxicology (IIT), Michigan State University East Lansing MI 48824 USA; k Department of Microbiology, Faculty of Science, Ain Shams University Cairo Egypt

## Abstract

The COVID-19 pandemic is the largest global public health outbreak in the 21^st^ century so far. It has contributed to a significant increase in the generation of waste, particularly personal protective equipment and hazardous medical, as it can contribute to environmental pollution and expose individuals to various hazards. To minimize the risk of infection, the entire surrounding environment should be disinfected or neutralized regularly. Effective medical waste management can add value by reducing the spread of COVID-19 and increasing the recyclability of materials instead of sending them to landfill. Developing an antiviral coating for the surface of objects frequently used by the public could be a practical solution to prevent the spread of virus particles and the inactivation of virus transmission. Relying on an abundance of engineered materials identifiable by their useful physicochemical properties through versatile chemical functionalization, nanotechnology offers a number of approaches to address this emergency. Here, through a multidisciplinary perspective encompassing various fields such as virology, biology, medicine, engineering, chemistry, materials science, and computer science, we describe how nanotechnology-based strategies can support the fight against COVID-19 well as infectious diseases in general, including future pandemics. In this review, the design of the antiviral coating to combat the spread of COVID-19 was discussed, and technological attempts to minimize the coronavirus outbreak were highlighted.

## Abstract 2

COVID-19 pandemic has significantly impacted waste generation in the form of personal protective equipment. This review article examines the nanotechnology applications to reduce the environmental risks of waste associated with the COVID-19 pandemic.

## Graphical abstract

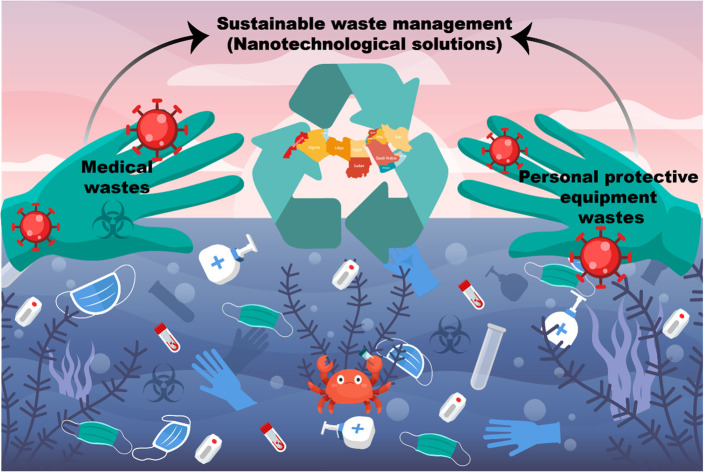

**Environmental significance**

The COVID-19 pandemic has significantly impacted waste generation, particularly in the form of personal protective equipment. Proper management of such waste is essential to prevent the spread of infectious agents and protect public health. This review article examines the potential applications of nanotechnology to reduce the environmental risks of waste associated with the COVID-19 pandemic.

## 1 Introduction

Since the emergence of Coronavirus disease (COVID-19), the threat of waste pollution has grown exponentially, with strong attention on the environmental and human health consequences of millions of personal protective equipment (PPE) (*e.g.*, face masks, shields, gloves, and wipes),^1–3^ and hazardous medical wastes (HMW) being used and discarded.^4,5^

Billions of PPE^6^ (*e.g.*, face masks, shields, gloves, and wipes) and HMW (Table 1) are generated globally every day, and these wastes may eventually enter the environment *via* disposal in landfills or littering. Disposable PPE wastes are mainly made of plastic polymers such as polypropylene, polystyrene, polycarbonate, polyethylene, or polyester, although other types of fabrics, such as cotton, are also used. The mass consumption of PPE has brought about wide concern over the generation of a huge amount of plastic waste, which is likely to become a source of secondary microplastics (MPs, < 5 mm) or even nano plastics (NPs, < 1 μm). Hospitals and laboratories, in particular, have generated increased amounts of hazardous medical waste due to the increased use of PPE and other single-use items. Proper waste management is essential to prevent the spread of infectious agents and protect public health.^7^

**Table 1:** Hazardous COVID-19 pandemic health care waste

Hazard	Example
Chemical waste	Laboratory reagents, film-developing reagents, solvents, expired/unused disinfectants, and waste containing heavy metals (batteries, broken thermometers, blood-pressure gauges, *etc.*)
Infectious waste	Materials contaminated with blood and body fluids, human excreta, laboratory cultures, and microbiological products PPE, *i.e.* boots, long-sleeved gowns, heavy-duty gloves, masks, goggles, and faces hields
Radioactive waste	Unused liquids from radiotherapy or laboratory research. Radioactive contaminated glassware, packages/absorbent paper, urine, and excreta from patients treated or tested with unsealed radionuclides also constitute radioactive waste
Sharps waste	Needles, auto-disable syringes, syringes with attached needles, infusion sets, scalpels, pipettes, knives, blades, and broken glasses
Pharmaceutical waste	Pharmacies, distribution centers, and hospital wastes
	Expired and contaminated pharmaceutical products

Several risks are associated with the waste generated during the COVID-19 pandemic.^4^ One of the primary concerns is the potential for waste to contribute to land and water pollution. This can occur when waste is improperly disposed of, for example, discarded in streets or waterways, or when it is not adequately contained or treated during the disposal process. This can lead to the contamination of soil and water sources and can have serious consequences for the environment and human health. Another risk associated with COVID-19 waste is the potential for exposure to contaminated materials. This can be a concern for waste workers and individuals who may come into contact with discarded PPE or other potentially contaminated materials.^8^ For example, discarded gloves or face masks could be touched or handled by individuals who are not wearing gloves, potentially leading to the transmission of the virus. According to the World Health Organization (WHO), the improper disposal of PPE can lead to the contamination of soil and water, as well as the potential transmission of the virus to others through the handling of discarded materials.^9^

Overall, the risk of waste associated with the COVID-19 pandemic highlights the importance of robust systems for managing waste and the need for ongoing efforts to minimize this waste's environmental and health impacts.^10,11^ Proper disposal and handling of COVID-19 waste are essential to minimize these risks and protect both the environment and public health.^4,5^

Nanotechnology is a promising approach for addressing the waste management challenges associated with the COVID-19 pandemic.^12^ One example of a nanotechnology-based approach for waste management is using nanocomposites,^13^ which combine a matrix material with nanoscale fillers. These materials can potentially improve traditional materials' properties, such as increasing their strength or decreasing their weight. In the context of waste reduction, nanocomposites could be used to develop more durable products, such as masks and gloves, which would reduce the amount of waste generated. Nanocomposite can also be used to develop biodegradable plastics, which would help reduce the environmental impacts of plastic waste.^14^ Other potential applications of nanotechnology for waste management include using nanocatalysts to enhance the efficiency of waste treatment processes and using nanosensors to monitor the quality of recycled materials and improve the efficiency of recycling systems. Overall, using nanotechnology in waste management holds significant potential for reducing the environmental risks associated with the COVID-19 pandemic.^15–17^

A massive research effort has been launched to understand, characterize, and estimate the exposure risks of PPE and HMW-associated contaminants. While the number of studies examining the impacts of PPE is increasing, the emerging impacts of the COVID-19 pandemic on the environment remain unelucidated, which is a significant knowledge gap requiring further investigation.

**Fig. 1:**
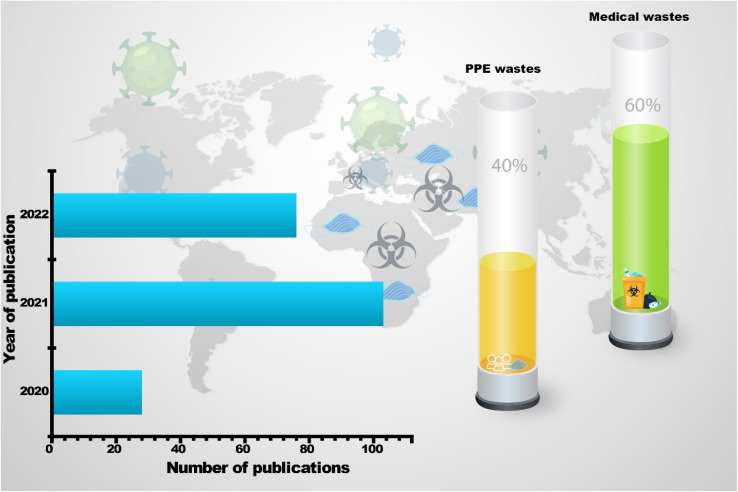
Number of publications related to waste associated with the COVID-19 pandemic between 2020 and 2022 (Scopus database 01-12-2022), and the percentage of each waste group.

According to SCOPUS data, there is increased growth in the number of publications in 2020–2022, demonstrating the broad interest given by the scientific community to waste associated with the COVID-19 pandemic. Moreover, few papers reported and discussed the nanotechnological approaches for waste management associated with the COVID-19 pandemic (Fig. 1).

In light of the current situation regarding Covid-19 disease, a discussion is proposed on the need for research focused on the presence and evolution of SARS-CoV-2 in water, soil, and other environmental compartments affected by the application of wastewater and sewage sludge. The evaluation of current wastewater and sewage sludge treatments, as well as the possible development of new specific techniques based on sorption, nanotechnology, *etc.*, would also be of great interest for controlling the environmental spread of these viruses in current and future epidemics.

## 2 Implications of COVID-19 on waste generation

Nzediegwu used the population data and reported the estimated total daily face masks for Egypt (≈70.4 M), Morocco (≈37.8 M), Algeria (≈51.2 M), and Tunisia (≈13.2 M).^18^ While Benson *et al.* reported the estimated face mask disposal for Algeria (≈22.4 M), Egypt (≈30.8 M), Libya (≈3.8 M), Morocco (≈16.5 M), Sudan (≈10.7 M), and Tunisia (≈5.9 M).^2^ The General Authority for Statistics of Saudi Arabia forecasted daily mask use to be 33.5 M.^7^ A large cross-section study (*n* = 6770) performed in Hello the MENA region during April and May 2021 showed a significant increase in thrown PPE on streets, rivers, and lakes in urban areas.^19^ Surveying health care workers in Qatar (*n* = 1757) showed that 49.7% complied with PPE use.^20^ Similarly, (45.16%, *n* = 98) were satisfied with their PPE in a Kuwaiti study.^21^ A clean-up campaign in June–August 2020 in Jeddah, Saudi Arabia, resulted in the collection of 848 plastic wastes and PPE, representing 49% of the total plastic items. The results indicated the presence of 0.86 PPE item m^−2^ in Jeddah, and PPE was increased significantly (*P* < 0.001) by 48% on weekends. Also, Hassan *et al.* suggested the presence of 0.29 and 2.79 PPE item m^−2^ in Hurghada and Alexandria. Interestingly, PPE was increased significantly (*P* < 0.001) by 76.3% in Alexandria during the weekends, while the increase in Hurghada was insignificant (7%).^22^ In Morocco, a survey involving 185 individuals from Salé-Kénitra Casablanca-Settat and Rabat-regions revealed that 70% used their PPE once and dropped them in household waste or trash bins^23^ – results from Tighassaline and Khenifra cities were comparable as 87% of participants mixed PPE with household waste.^3^ The Mejjad *et al.* survey suggested using and disposing of five million face masks daily in Casablanca-Settat and Rabat-Salé-Kénitra, which is almost 35% of the total Moroccan-engendered face mask waste.^23^ Also, Haddad *et al.* collected 689 PPE (face masks, 96.81%) from the Agadir coastline in Morocco and showed an average density of 1.13 × 10^−5^ PPE m^−2^.^6^ The average daily PPE weight for five health care facilities in Bahrain was 1849.^5^ Most PPE in the Caspian Sea (Iran's coast) in Mazandaran province were face masks (95.3%). The average PPE density was 1.02 × 10^−4^ PPE m^−2^ (range, 0.00 to 7.16 × 10^−4^ PPE m^−2^).^24^

**Fig. 2:**
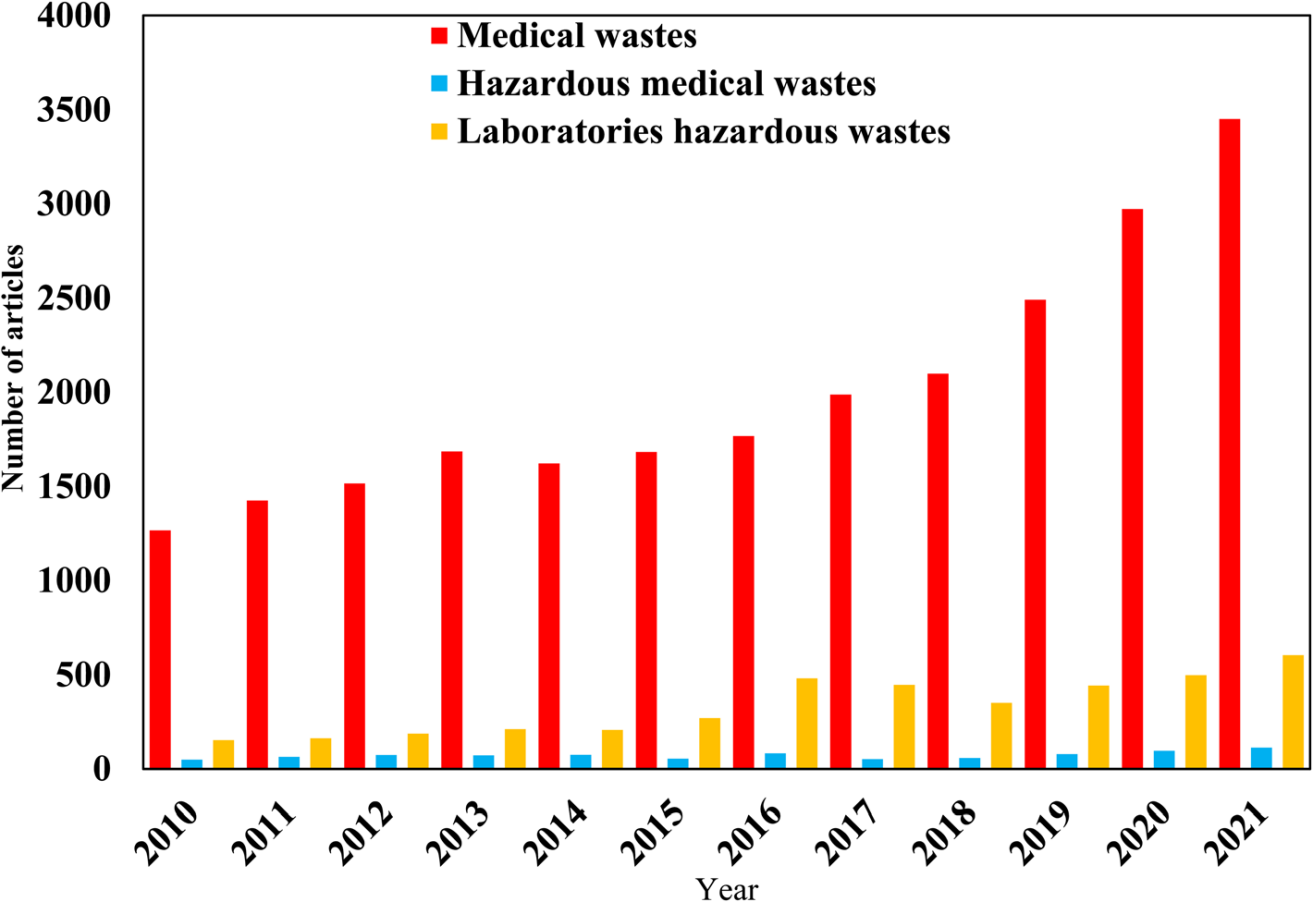
Literature on Hospital hazardous wastes, Hazardous medical wastes, and Laboratories hazardous wastes topics by web of science (a tool from Clarivate Analytics, 2020) since 2010, analyzed by years.

Additionally, Web of Science (a tool from Clarivate Analytics, 2022) was searched (September 03, 2022) using the keywords of “Medical wastes, Hazardous medical wastes, and Laboratories hazardous wastes” during the last twelve years from 2010 to 2021 (Fig. 2). In this regard, and as it is a serious environmental concern, several works have been carried out for MW safe treatment.^10,25–30^

## 3 Risks of waste associated with COVID-19

**Fig. 3:**
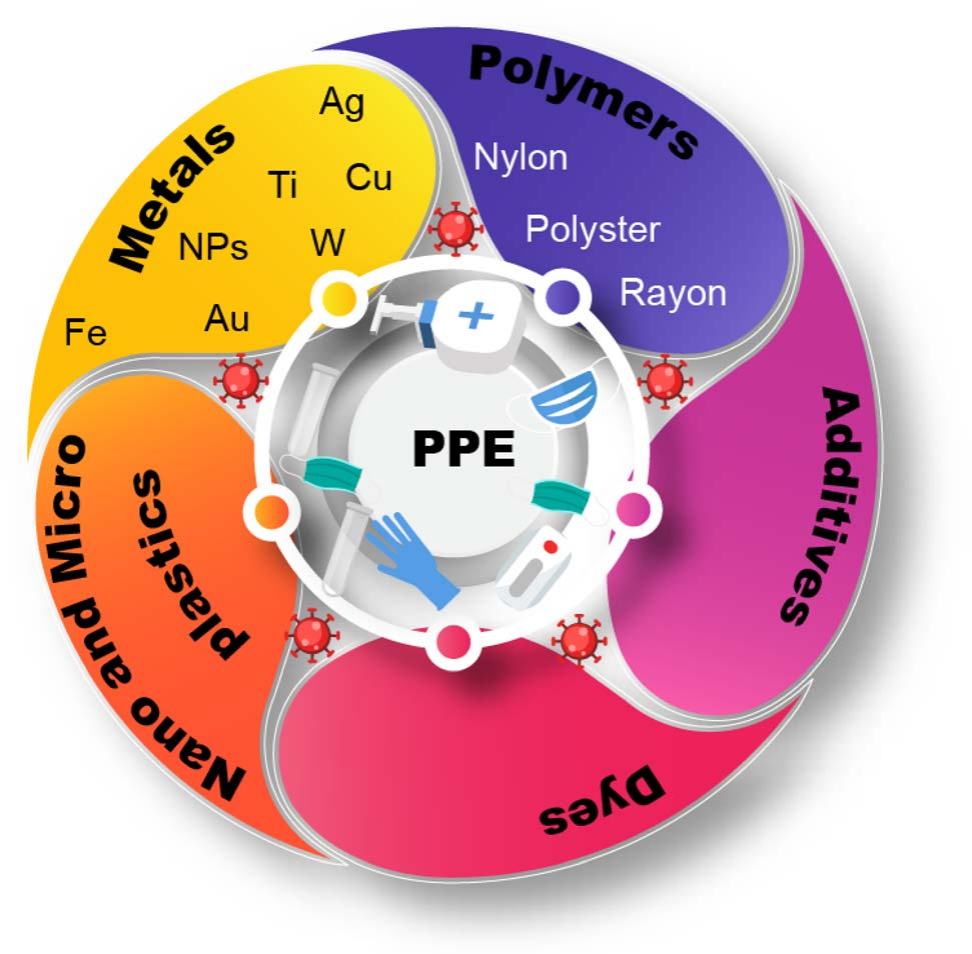
Contaminants associated with PPE.

**Fig. 4:**
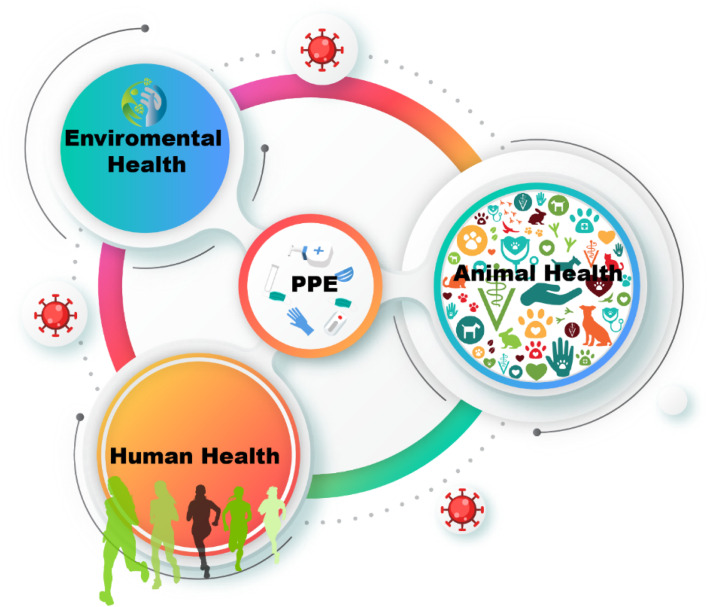
Potential effect of PPE on the environment, animal and human health.

Excessive PPE use has generated many contaminants (Fig. 3). Subsequently, all the wastes released are also likely to cause serious harm to the environment, animals, and human health (Fig. 4). Polypropylene is commonly used in N-95 masks, whereas Tyvek is used in medical face shields, suits, and protective gloves. Microplastics have a long life span and can pollute the environment with dioxin and other toxic substances.^31,32^

**Fig. 5:**
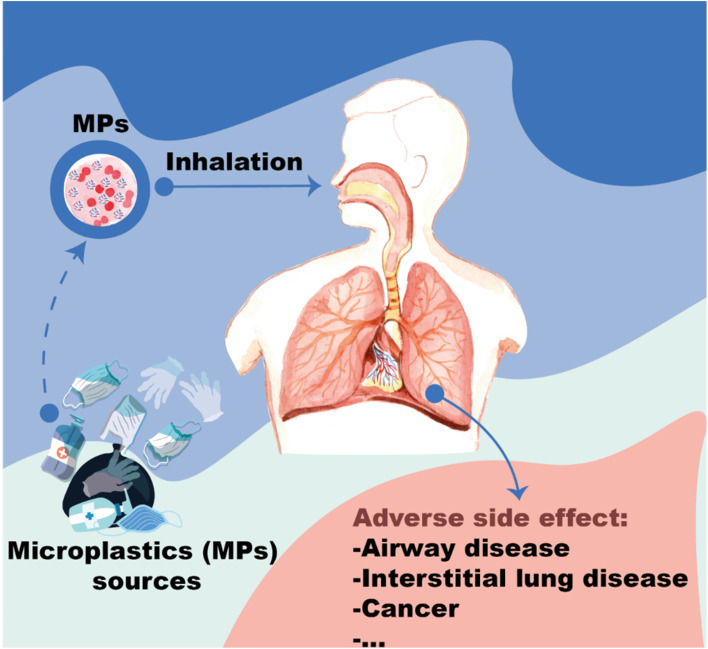
Risks of micro-plastics and nano-plastics on human health.

Three layers make up surgical masks. The front and back are made of polypropylene and have fibres that are around 20 m in diameter.^1,33^ The core layer of surgical masks is made of melt-blown fabric, which is made from melt-blown fabrics made of polypropylene, one of the major components.^34^ In general, melt-blown fabrics exhibit high filtration performance, allowing them to eliminate bacteria, suspended particles, droplets and aerosols because their fiber diameters are approximately 1–5 m. The melt-blown filter, which is produced by conventional micro- and nanofiber manufacture, is the main filtering layer of the mask. Molten polymer is extruded *via* small nozzles with high-speed blowing gas in this process.^35^ As a result of using and reusing masks made from the aforementioned materials, microplastics and nano plastics can be produced.^1,33^ Breathing under these conditions can result in microplastic inhalation. It is also possible to breathe in microplastics while breathing the air^36,37^ (Fig. 5). Abbasi *et al.*^38^ detected 900 microplastics and 250 microrubbers of various sizes and shapes in 15 g of street dust collected in Iran.^38^

Lai *et al.*^39^ show that wearing a mask increases the danger of breathing microplastics, particularly microfibers.^39^ One should also consider inhaling microplastic from the air to protect human health. In this situation, masks serving as a barrier may help to minimize the amount of microplastic inhaled while breathing.^40^ Lai *et al.*^39^ proposed that even when masks are worn continuously for 720 hours, they greatly reduce the risk of breathing particles. Tables 2 and 3 show the total quantity of fiber-like microplastics and particles observed collected throughout the 720 hours breathing simulation test while wearing various types of masks.^39^

**Table 2:** The total quantity of fiber-like micro-plastics that were collected throughout the 720 hours breathing simulation test while wearing various types of masks

Time (h)	No mask	Surgical-A	Surgical-B	Fashion	Non-woven	Cotton	Activated carbon	N95
2	172	38	112	69	47	92	135	25
4	197	57	138	83	69	123	249	45
8	237	91	214	133	115	180	447	80
24	275	137	264	185	150	222	540	110
48	348	202	374	245	211	303	725	179
96	428	301	516	397	341	484	958	268
120	482	392	620	478	418	581	1120	308
168	643	515	780	654	540	741	1352	366
360	911	961	1556	1318	1026	1337	2086	719
720	1835	1913	3180	2576	2134	2824	3984	1521

**Table 3:** The total quantity of particles observed that were collected throughout the 720 hours breathing simulation test while wearing various types of masks

Time (h)	No mask	Surgical-A	Surgical-B	Fashion	Non-woven	Cotton	Activated carbon	N95
2	3918	1808	3090	3110	2152	2241	2212	1695
4	7946	2648	6568	6622	3612	3567	3417	2268
8	15.732	3797	12.625	12.158	6292	6963	6033	3290
24	39.700	6631	24.279	24.643	7814	12.848	9988	4678
48	92.236	10.495	42.119	47.722	14.598	26.690	16.174	6790
96	184.618	22.081	76.833	91.800	25.705	46.892	29.912	9294
120	212.994	26.585	99.487	109.986	29.269	55.932	34.814	10.810
168	249.114	36.953	128.054	145.374	36.423	72.006	47.028	12.660
360	562.842	71.545	268.897	295.832	85.664	158.660	92.453	23.265
720	1.121.316	140.069	523.791	597.980	169.316	302.242	181.017	44.853

Additionally, Fish and marine species exposed to treated wastewater have been found to have plasma levels of ketoprofen, ibuprofen, naproxen, and diclofenac.^41^ Spilled alcohol in water harms aquatic life, and spilled alcohol on soil can contaminate groundwater.^31^ Our water systems, soil, and biota will suffer long-term effects from this unwelcome set of events.^32,42^ Excessive use of soap as a preventive strategy for COVID-19 results in a massive concentration of soap in wastewater, which can harm aquatic plants. A 120 mg L^−1^ of soap can stop algal formation and proliferation. Plants like *Potamogeton* and *Ranunculus aquatilis* cannot flourish at detergent concentrations of 2.5 ppm.^43^ The soil quality may be affected by the accumulation of toxic chemicals in the soil brought on by heavy soap use. Domestic waste contaminates rivers, which contaminates lakes and oceans.^42^

**Fig. 6:**
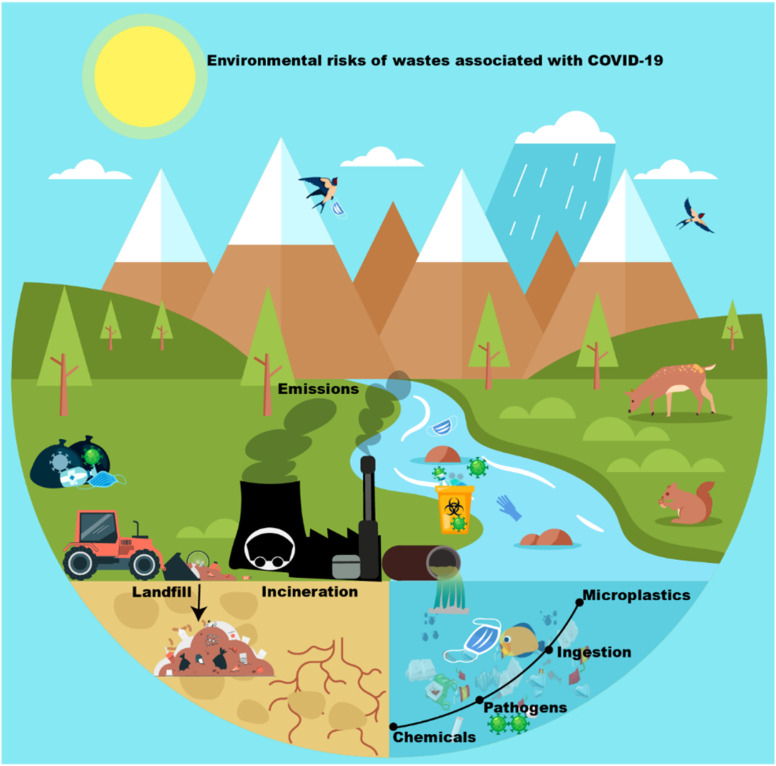
Environmental risks of wastes associated with COVID-19.

It is worth mentioning that adequate waste management may solve occupational hazards to a large extent. Another way, such as incineration, is considered one of the available solutions and widely practiced medical waste disposal. Nevertheless, incineration of medical wastes releases pollutants into the air and ash residue. Also, the incineration of materials that contain heavy metals like lead, mercury, and cadmium caused an increment in their concentrations, while some materials that contain dioxins, furans, and metals accumulate in the environment. Thus, materials containing chlorine, dioxins, and furan or metals should not be incinerated because it considered human carcinogens^44^ (Fig. 6).

## 4 Waste management strategies during the COVID-19 pandemic

During COVID-19, environmental health awareness and waste management have become more prominent since generating gigantic and infectious health-care waste (HCW) quantities without proper treatment, control, and management could increase the widespread outbreaks probability of virus infection.^45^ The first and most important guideline provided by numerous member states of the EU, is increasing the capacity for each country to manage HCW due to the vast quantities of HCW during the pandemic. The HCW should be stored in sealed packages and containers in specially protected areas that only allow authorized personnel to enter. Disinfectants such as chlorine must be used on the outer and inner packages and containers to avoid the possible transmission of the virus. Furthermore, all the staff members, collectors, and authorized workers must be follows safety measures.^46^ Generally, lacking or incomplete, vague, and inconsistent information adds difficulties and uncertainty in waste management strategies decision-making.^45^ However, the challenges in underdeveloped African countries are even more significant due to less budget for WM compared to the other developed countries.^47^ Some improper WM strategies in some countries exacerbate the COVID-19 spread. India generates ≈517 tons of Biomedical Waste (BMW) per day, but only 501 tons per day can be treated in the 200 BMW treatment facilities spread over 28 states. While some states do not have these treatment BMW facilities at all.^48^ The garbage trucks in Tehran are principally equipped with compactors which facilitate the increase of waste collection. However, the high moisture content of the collected wastes generally results in overflowing the leachate from garbage trucks all over the road and in the landfill.^46^ In Tehran, followed the same WM strategies that were instructed by some cities in the USA during the COVID-19 outbreak, legal separation and recycling of municipal solid wastes have been completely stopped by the government due to concerns about the risk of COVID-19 spreading at transfer stations and recycling centers, while the illegal separation and recycling of wastes is still ongoing.^46^ Other governments, such as Italy, believe that waste separation is crucial during COVID-19 and that only households with infected individuals should be exempted. For example, Italy has only banned separating or recycling waste from infected residents.^49^

There are many reasons for the failure of adequate WM strategy in several countries:

• Inability to raise awareness about the health hazards related to HCW.

• Inappropriate training for adequate waste management.

• Absence of regular WM and disposal systems.

• Insufficient financial and human resources.

• Absence of proper regulations and rules in many countries or do not enforce them.

• Absence of surveillance system.

More attention and diligence should be focused on HCW management to overcome these challenges to avoid adverse health outcomes, including exposure to infectious agents and toxic substances associated with poor practice.

WHO has introduced the first universal and inclusive guidance document for safe WM from health care activities, including the regulatory framework, planning issues, handling, waste minimization, recycling, storage, transport, treatment, and disposal options.^50^ Strategies instructed by the WHO to improve HCW management:

• Supporting the practices that increase the amount of generated HCW and ensure proper waste separation.

• Improving systems and strategies as well as strong monitoring and regulation to progressively improve the practices of waste segregation and disposal practices to meet national and international standards and be easy to follow.

• Preferring the safe and environmentally sound treatment of hazardous health care wastes (*i.e.*, by autoclaving, microwaving, steam treatment, and chemical treatment) over incineration. Also, selecting safe and environmentally friendly management options through all HCW management processes, to protect humans from hazards or danger when, handling, collecting transporting, storing, treating or disposing of waste.

• Building a comprehensive HCW management system, addressing responsibilities, resource allocation, handling, recycling, storage, transportation, treatment, and disposal. This is a long-term process, sustained by gradual improvements from the government's commitment; raise of consciousness about the risks associated with health care waste and safe handling practices.

## 5 Proposed approaches for waste management associated with the COVID-19 pandemic: nanotechnological solutions

The world is facing the current COVID-19 pandemic, which points to the urgent need to develop new medical innovations, technologies, or innovative strategies to fight COVID-19 infection and adapted solutions to manage the waste generation due to the coronavirus pandemic.^2^ The coronavirus infection route is still poorly understood and appears to be spread by air, surface, and water. Controlling the ways of surface transmission of coronavirus may be promising in reducing the spread of the infections in public, indoor, and outdoor environments.^15,51^ Developing antiviral coatings based on nanotechnological approaches may be one of the most prominent ways to counter viral transmission.^52–59^ It could be a potential strategy for practical applications, not only in hospitals for clinical purposes but also to control the spread of coronaviruses and inactivate any possible contamination.^14^ Accordingly, the entire surrounding environment must be disinfected at all times.^9^ To achieve this, nanomaterials with excellent antiviral properties should be sought that are cost-effective, environmentally friendly, and practically applicable.^51^

This section discusses several approaches to waste management associated with COV-19, based on nanotechnology solutions.

### 5.1 Antiviral coatings

Nanomaterial-based antiviral surfaces or coatings that can work effectively in both indoor and outdoor environments might be more prospective for such purposes, as the infectious viruses are located in close proximity to patients in hospitals at home or in common public areas. In this regard, photocatalytic surfaces could be more promotive as they permanently inactivate, oxidize and destroy micro-organisms such us viruses and bacteria under normal ambient lighting conditions, *i.e.*, they are also effective in an indoor environment.^13,52,60,61^ Recent studies point to the tremendous potential of photocatalytic material surfaces-based nanomaterials for the inactivation of coronaviruses' (Table 4).^60,62^

**Table 4:** Antiviral coatings for COVID-19 degradation

Materials	Outcomes	Reference
TiO_2_/Ti photocatalyst coating	Significant antiviral activity, with a decrease rate of virus reached 99.96% for influenza virus and 99.99% for SARS-CoV-2	71
water-borne polymer coating	SARS-CoV-2 variants inactivation within 30 minutes of exposure	88
Ag–TiO_2_ nanocomposite coatings on ceramic tiles	Inactivate SARS-CoV-2 under ambient indoor lighting, with an 87% reduction in titers at 1 h and total loss at 5 h of exposure	67

Titanium-based structures are among the most often used photocatalysts due to their high photo-oxidation of organic compounds, superior chemical stability, high oxidizing power under UV light, and outstanding chemical resistance and photostability. TiO_2_ has also been shown to have the ability to eradicate both Gram-positive and Gram-negative bacteria, as well as different viral types and parasites.^58^

**Fig. 7:**
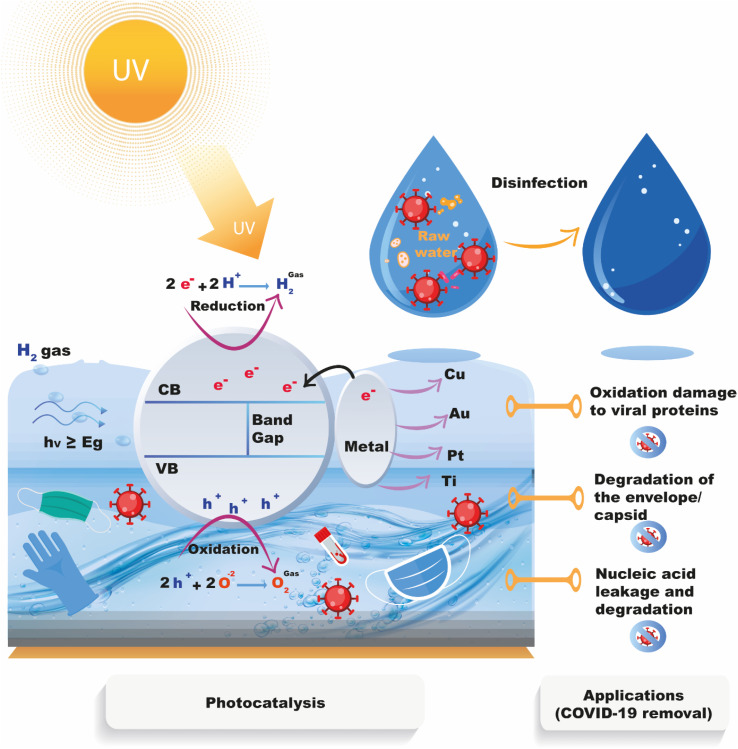
Mechanisms of photocatalytic inactivation of coronavirus for water treatment application.

TiO_2_ has been the focus of much interest these last years in the domain of microbial remediation by photocatalysis due to its potent photocatalytic antimicrobial action against various types of microbes. Under exposure to oxygen and UV light, water decomposes on the surface of TiO_2_ into ROS which act as oxidizing or reducing agents causing the decomposition of organic and microbial materials. In addition, TiO_2_ has proven to be very useful in visible light activity in both indoor and outdoor applications for environmental disinfection. This photocatalytic disinfection effect has also shown great promise in controlling various viruses as an antiviral photocatalyst.^63^ The ROS formed at the surface of TiO_2_ (˙OH and O_2_^−^˙) due to UV activation, have good performance to degrade the capsid and or the envelope proteins of non-enveloped viruses as well as the phospholipids of the enveloped ones.^64^ In addition, due to the resulting leaching and degradation of nucleic acids, the virus particles eventually become inactive, as shown in Fig. 7.

Former studies demonstrated that the illumination of TiO_2_-based photocatalysts allow to obtain high added value oxidizing/reducing free radicals with outstanding antiviral action against various viruses such as the COVID-19 virus which is aerosol transmitted and leads respiratory tract infection.^65,66^ Evidence shows that TiO_2_-based nanomaterials have potential properties to combat COVID-19.^66–68^ Recently, Khaiboullina *et al.*^66^ proved that a photosensitive coating based on TiO_2_ NPs under exposure to UV light may be employed as an effective way against the spread infection by COVID-19 substitutes and could play a major role in the destruction of viral particles. Using the same photocatalyst material Micochova *et al.*^67^ investigated the stability of SARS-CoV-2 virus using thin Ag–TiO_2_ nanocomposite coatings on ceramic tiles under ambient light conditions. The viral titer was reduced by 4 orders of magnitude, reaching a complete inactivation after 20 minutes of light exposure and no detectable active virus was found after 5 hours of treatment. Importantly, SARS-CoV-2 on uncoated tiles was fully infectious after 5 hours of virus's addition. Overall, tiles coated 120 days earlier were able to inactivate SARS-CoV-2 under ambient indoor lighting, with an 87% reduction in titers at 1 h and total loss at 5 h of exposure.

Mathur *et al.*^69^ provided evidence regarding the UV destruction mechanism of TiO_2_ that can provide effective results in air purification by destroying or inactivation of various microorganisms such as bacteria and pathogenic viruses. It was suggested that this model could be promising for various air conditioning systems to make free cabin from bacterial and viral infections. Recently Uppal *et al.*^70^ proposed TiO_2_ photocatalytic coating for virucidal activity against HCoV-OC43 virus which is a member of beta coronavirus family just like SARS CoV-2 under the influence of UV irradiation. It was found that the TiO_2_-coated glass surface exhibited good antiviral response against the virus compared to uncoated glass using T-qPCR and virus infectivity tests. The results show a strong reduction in viral RNA copies and viral infectivity with increasing exposure time, reaching complete disinfection within 60 minutes of UV exposure.

**Fig. 8:**
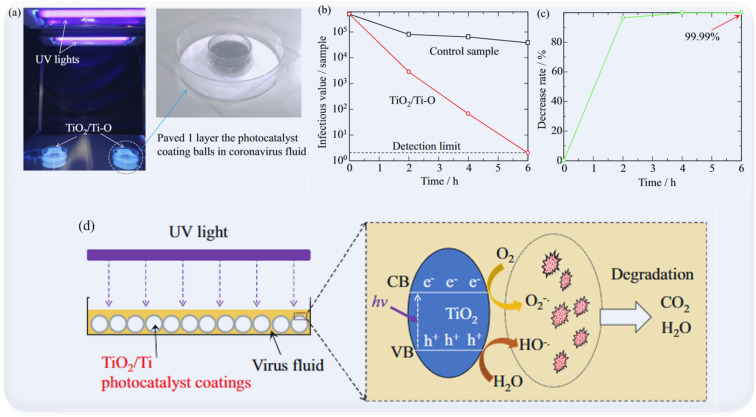
The inactivation test for SARS-CoV-2 of the TiO_2_/Ti–O sample. (a) The set-up, (b) the infectious value change of SARS-CoV-2, (c) the decrease rate of SARS-CoV-2, (d) proposed mechanisms of viral inactivation induced by TiO_2_/Ti photocatalyst coatings. Adapted from https://www.nature.com/articles/s41598-022-20459-2.

Various TiO_2_ nanostructures such as nanotubes, nanoparticles, and nanowires, have also been researched and developed to enhance antibacterial and antiviral photocatalysis to inactivate viruses, including the COVID-19 virus, with high efficiency. Negrete *et al.*^62^ demonstrated an excellent photocatalytic effect of Ag–TiO_2_ nanomaterials to eliminate SARS-COV-2 after exposure to UV light for 9 h. Similarly, Hamza *et al.*^68^ investigated the effect of TiO_2_ NTs for disinfection of SARS-CoV-2 which showed strong anti-SARS-CoV-2 activity at very low cytotoxic concentrations *in vitro* as well as excellent antiviral activity at a very low concentration (IC_50_ = 568 ng mL^−1^) showing the relevance of TiO_2_ nanostructures as a coating nanomaterial with potent disinfectant properties to combat SARS-CoV-2. Interestingly, Matsuura *et al.*^65^ evidenced that TiO_2_ coating could be effective in inactivating SARS-CoV-2 *via* time-dependent TiO_2_-mediated photocatalytic reactions. From the findings, the photocatalytic reaction induced by TiO_2_ nanostructures showed good results in removing SARS-CoV-2 from contaminated water. Recently, Lu *et al.*^71^ developed a TiO_2_ photocatalyst coating on aluminium oxide (Al_2_O_3_) balls that may successfully inactivate influenza and SARS-CoV-2 viruses. The coating was discovered to be non-toxic and chemically stable, and it may be applied in working environments without requiring people to evacuate. The coating, according to the researchers, might be employed in air filtration systems to help reduce the spread of airborne viruses. According to the findings, photoelectrochemical oxidation-aided air purifiers might be useful instruments for reducing indoor SARS-CoV-2 exposure. Because of their strong decomposition function for C_2_H_4_O and CH_2_O, the TiO_2_/Ti photocatalyst coatings were also shown to be useful for environmental purification. Importantly, TiO_2_/Ti photocatalyst coatings are capable of considerable viral inactivation, with 99.96% inactivation for influenza virus and 99.99% inactivation for novel coronavirus (Fig. 8).

**Fig. 9:**
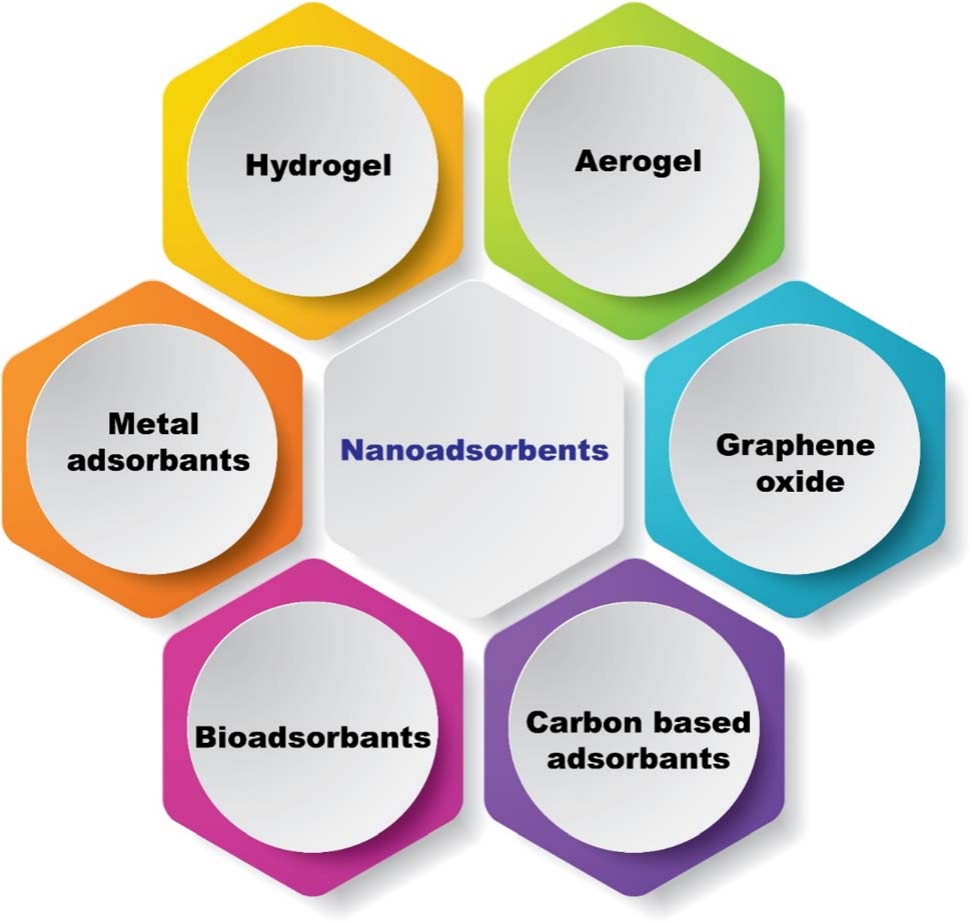
Nano-adsorbants for COVID 19 removal.

Various metal, nanocomposite, and semiconductor materials have been utilized to engineer antibacterial as well as antiviral coating surfaces for the deactivation of viruses or bacteria.^57,72–83^ Likewise, diverse surfaces-based nanomaterials consisting of metals, semiconductors, and alloys have been applied to fight COVID-19.^52,53,58,84–87^ Versatile nanomaterial-based antiviral surfaces or coatings that can work effectively outdoors and indoors would be more promising in this regard, as the most infectious viruses are found close to patients in hospitals or in their homes. In this sense, nano-adsorbents for COVID-19 removal, such as aerogel, graphene oxide, hydrogel, carbon-based nanomaterials, and metallic adsorbents (Fig. 9), might be more promising because they remove viruses permanently and induce their inactivation from the environment. Metal nanoparticles and ionic species were discovered as potential materials to fight COVID virus.^58^ The interaction mechanisms between nanoparticles and COVID-19 can be divided into two main groups. In indirect interactions, nanoparticles do not directly affect viruses. However, they enhance the antiviral activity of the drug. An indirect mechanism uses nanoparticles to deliver antiviral agents to increase stability and improve bioavailability.^58^ Recently, graphene was used as a nano-adsorbent and developed a self-cleaning mask using a laser-induced dual-mode forward transfer method to deposit several graphene layers on a non-woven mask. Super hydrophobicity is confirmed on the graphene-coated mask surface, and incoming water droplets bounce off the mask surface. Since the surface temperature of the mask rapidly reaches 80 °C or higher in direct sunlight, the mask can be reused by sterilizing it in the sun. While ordinary masks absorb only a small amount of sunlight, graphene-coated masks absorb over 95% of the entire sunlight spectrum from 300 to 2500 nm. As SARS-CoV-2 is heat sensitive, the development of graphene-coated photo-thermal masks with promising self-sterilization capabilities holds great promise for large-scale production of effective PPE to combat coronavirus.^87^

**Fig. 10:**
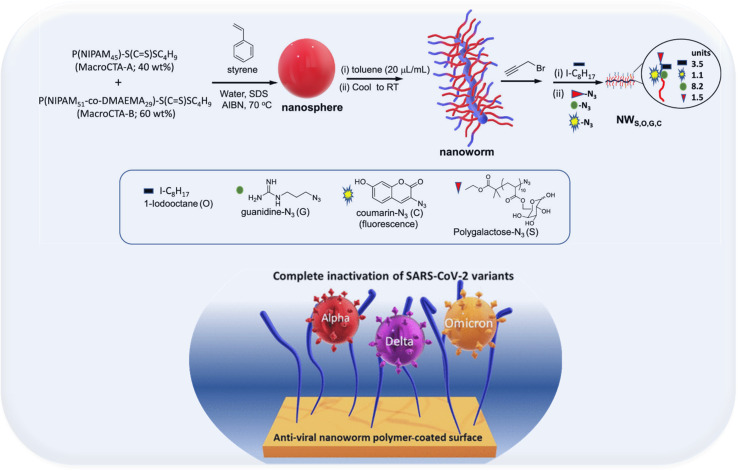
Synthesis of the functional nanoworms through the Environmentally Friendly Emulsion Polymerization Method and Post-Modification with Chemically Functional Groups for Capturing (Polygalactose) and Rupturing (Octane Groups) of SARS-CoV-2, Degradation of its mRNA (Guanidine Groups), and Detection of Polymer Coating (Coumarin Groups) adapted from Surface Inactivation of Highly Mutated SARS-CoV-2 Variants of Concern: Alpha, Delta, and Omicron|Biomacromolecules (https://acs.org).

Recently, polymeric coatings have been investigated to combat COVID 19. In this regard, Bobrin *et al.*^88^ designed an environmentally friendly polymeric water-borne nanoworms coating that can completely inactivate the highly mutated variants of SARS-CoV of concern: Alpha, Delta and Omicron, independent of the infectious variant (Fig. 10). The coating can be applied to masks and many other surfaces to capture and inactivate the virus, helping to reduce the transmission of SARS-CoV-2 and the evolution of new variants of concern. The polymer was developed to target the highly glycosylated spike protein on the surface of the virion and to inactivate the virion through nanomechanical disruption of the viral membrane. The results reveal that even with minimal levels of surface coating (1 g m^−2^), alpha, delta, and omicron viruses were completely inactivated, and their viral genome was degraded. In addition, the results reveal that the polymer causes little or no skin sensitization in mice and is non-toxic when consumed orally in rats.

### 5.2 Nanofiltration solutions

**Fig. 11:**
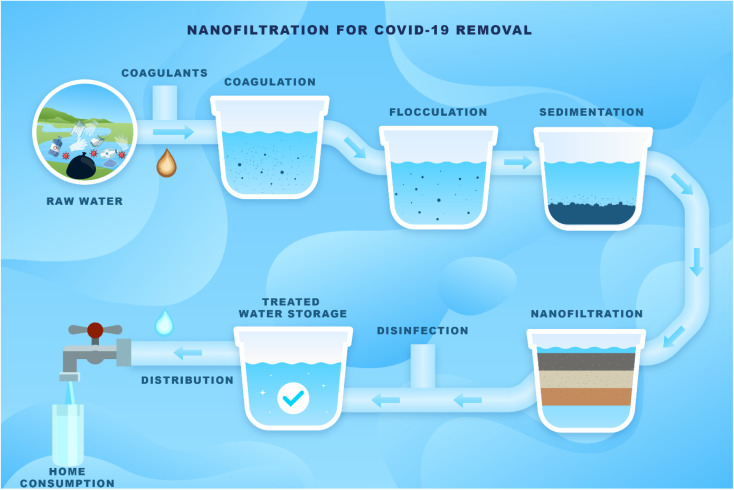
Nanofiltration solution for COVID-19 removal.

The most common pathway of transmission of respiratory diseases is by direct contact or indirect exposure to particles and droplets containing viruses.^89^ Effective elimination of these droplets is achieved through antiviral nanofiltration (Table 5, and Fig. 11). Recently, Issman *et al.*^90^ proposed an antiviral air filter using CNTs, which are mechanically reinforced by permeable and porous polyester with approximately 100 mm holes to improve filtration efficiency and structural stability. For this purpose, through an easy and continuous procedure, CNT aerogels were spun onto the support, and a bilayer hybrid is formed, a thin CNT membrane on a 0.4 mm thick, porous polyester. The prepared air filter showed 99.9% filtration efficiency. The electrically conductive feature of this air filter can be instantly heated to 130 °C in seconds for complete inactivation or removal of viruses on the filter surfaces, such as beta-coronavirus.^90^ Given the rapid spread of the Delta and Omicron variants of COVID-19, which pose a significant global public health risk, Cano-Vicent and collaborators investigated the cytotoxicity and antiviral action of calcium alginate films to inactivate COVID-19 enveloped virus.^12^ Calcium alginate biomaterial films demonstrated no cytotoxicity effects on human keratinocytes showing good antiviral performance against enveloped viruses, including Delta variant of SARS-CoV-2 and the bacteriophage phi 6, with 96, and 94% inactivation, respectively. The high antiviral effectiveness was related to the negative charge density of the alginate polymer network that binds to viral envelopes by inactivating their membrane receptors.

**Table 5:** Nano-filters for virus removal

Materials	Outcomes	References
Alginate/copper sulfate	Effective against COVID-19 by 99.99%	91
	Low cytotoxicity	
Polypropylene/tannic acid	High-performance filtration (up to 2730 pfu mm^−2^)	92
	Speedy filtration process (10 minutes)	
Cellulose fibers modified multi-walled carbon nanotubes and phenol-formaldehyde	0.64% of filtration efficiency	93
	High air permeability	
CNT on porous polyester	99.999% of filtration efficiency	90

An essential strategy for preventing coronavirus high spread is the use of masks. However, masks quickly become contaminated after use. Bataglioli *et al.*^91^ designed a coating textil surface based on copper sulfate/alginate on disposable masks for COVID-19 inactivation. The advantages of the developed new material are the use of inexpensive materials, processed using simple and affordable methods to allow easy scaling of an effective antiviral coating. The designed disposable face masks demonstrated an efficacy of 99% against coronaviruses, with low or neglect cytotoxicity to L 929 cells.^91^

## 6 Conclusions and recommendations for further research

The daily production rate of different types of COVID-19-related PPE and hazardous medical wastes are increasing significantly due to the high pandemic rate. A crowd of many thousands of people has been previously affected by the Covid-19 respiratory infection in several areas in the MENA region and the world. Indeed, it should be kept in mind that symptomatic and asymptomatic patients can spread coronavirus through their excreta, As they spread through the environment, some potential steps to consider for these viruses are moved from one compartment to another, entry into living beings, proliferation, possible mutation, transmission, *etc.* Currently, these coronaviruses are thought to survive in the environment outside of living cells for only a few days, which is enough to reach other organisms and mutate, change their properties, *etc.* It's possible that we should consider several possible scenarios in the near future. These possible short-term considerations should be considered as promptly as possible, in addition to previously aggressive measures to control direct airborne and indirect spread through other environmental vectors. Proper disposal of medical waste can therefore add value to the economy for sustainable-development in the MENA region and beyond. Additionally, it helps reduce the large-scale spread of the COVID-19 virus and other viruses in a given location. In addition, the efficacy and consequences of wastewater and sludge treatment and the potential for further diffusion into environmental compartments should be considered. Additional research will also examine the potential engineered treatments to retain and/or inactivate this virus and other pathogens circulating in such ecosystem compartments before and after their release, including sorbents and other materials, must be guessed. Nanomaterials such as metal oxide nanostructures, graphene, carbon nanotubes, carbon quantum dots, and titanium dioxide, as well as bio-nanoparticles such as chitosan, capped silver, graphene, gold, and silicon nanoparticles, are thought to play a key role in the development of antiviral coatings. While creating prospective coating materials, some of the most important things to examine are ease of use, low toxicity, health concerns, long-term efficiency, and sustainable manufacturing. The globe is in a difficult period, and offering a single solution for all sorts of surfaces is difficult, but it is feasible to build unique surface coating solutions based on already existing research studies. The multidisciplinary collaboration would undoubtedly aid the rapid development of antiviral surface coating materials. Furthermore, additional research to develop eco-friendly disinfection techniques based on nanotechnology that can potentially remove viruses from the environment without the effects of chronic exposure on other species.

Finally, an accurate, inexpensive, affordable, and straightforward to use on-site tool for monitoring COVID-19 in soil, water, and air, as well as to soften the socio-economic impact of COVID-19. We need to develop a biosensor. COVID-19 pandemics and other undesirable impact on the environment, human and animal health. The SARS-CoV-19 pandemic may be over by the end of 2022, but we must keep a watchful eye on future large-scale pandemics.

## Author contributions

GSE suggested the research topic, conceptualization, investigation, writing—original draft, visualization reviewing and editing. DE suggested the research topic, conceptualization, investigation, writing—original draft, visualization, editing and figure drawings MSG suggested the research topic, conceptualization, investigation, writing—original draft, visualization reviewing and editing. DME suggested the research topic, conceptualization, investigation, writing—original draft, visualization reviewing and editing. MA suggested the research topic, conceptualization, investigation, writing—original draft, visualization reviewing and editing. HGN suggested the research topic, conceptualization, investigation, writing—original draft, visualization reviewing and editing. MSK suggested the research topic, conceptualization, investigation, writing—original draft, visualization reviewing and editing. MAG suggested the research topic, conceptualization, investigation, writing—original draft, visualization reviewing and editing.

## Conflicts of interest

The authors state that the study was conducted without commercial or financial relationships that could be construed as a potential conflict of interest.

## Supplementary Material
